# Revitalizing Muscle Regeneration: Cocoa Polyphenols Shield Mitochondrial Integrity and Boost Myogenesis Under Oxidative Stress

**DOI:** 10.1002/jemt.24755

**Published:** 2024-11-30

**Authors:** Jose Angel Garcia‐Merino, Vittoria Carrabs, Fabio Ferrini, Sara Salucci, Michela Battistelli, Sabrina Burattini, Francesca Luchetti, Maria Gemma Nasoni, Giosuè Annibalini, Matteo Micucci, Federico Gianfanti, Piero Sestili, Mar Larrosa, Elena Barbieri

**Affiliations:** ^1^ Faculty of Health Sciences Universidad Europea de Canarias La Orotava Spain; ^2^ MAS Microbiota Group, Faculty of Biomedical and Health Sciences Universidad Europea de Madrid Villaviciosa de Odón Spain; ^3^ Departamento de Farmacia, Facultad de Ciencias de la Salud Universidad CEU Cardenal Herrera Valencia Spain; ^4^ Department of Biomolecular Sciences University of Urbino Carlo Bo Urbino Italy; ^5^ Institute of Oncology Research Bellinzona Switzerland; ^6^ Università della Svizzera Italiana Lugano Switzerland; ^7^ Department of Biomedical and NeuroMotor Sciences (DIBINEM) University of Bologna Bologna Italy

**Keywords:** C2C12 cells, mitochondria, muscle regeneration, nutraceuticals, oxidative stress

## Abstract

In this study, we describe the effect of cocoa polyphenol extract (CPE, from flavanols‐rich cocoa) on myogenic differentiation in murine myoblasts (C2C12 cells) exposed to H_2_O_2_. The myogenic program was monitored using morphological, ultrastructural, and molecular approaches. Treatment with 100 μM of H_2_O_2_ for 1 h decreased cell viability. C2C12 (D1) exposed to H_2_O_2_ shows more apoptotic and necrotic cells, and mitochondria appear emptied, with cristae heavily damaged. To evaluate the effect of CPE on myoblast viability and myotube formation, 10 μg/mL of CPE were added 24 h prior to H_2_O_2_ treatment and cells were supplemented with fresh CPE every 24 h during differentiation. Supplementation with CPE protected C2C12 myoblasts from H_2_O_2_‐induced oxidative damage both at early (D1) and late (D6) phases of differentiation, preventing cell death and mitochondrial damage. The number of mitochondria (per area of cell surface) increased 2‐fold in both control and in CPE‐supplemented and mitochondria in myotubes D6 showed a greater extension of mitochondrial cristae than mitochondria in D1. At D1 and D6 the monolayers showed surface and inner cell features relatively comparable to the untreated control suggesting that CPE supplementation significantly mitigated the effect of H_2_O_2_. Preliminary data obtained by the myogenic index (Giemsa staining) suggested that CPE‐supplemented cells were partially protected from H_2_O_2_‐induced myogenesis inhibition. The CPE supplementation seems to preserve the mitochondrial integrity and the myogenic differentiation ability of oxidatively injured C2C12 ensuing further nutraceutical perspectives.

## Graphical abstract

Experimental design and CPE treatment conditions. Differentiating myoblasts at D1 were treated with 0.1 mM of H_2_O_2_ for 1 h given 24 h following the differentiation commitment. To evaluate the effect of CPE on myoblast viability and myotube formation, 10 μg/mL of CPE were added at the beginning of the differentiation (D0), then the medium was changed with fresh CPE every 24 h. C2C12 cells were analyzed after acute (D1) and chronic (D4, D6) CPE supplementation.

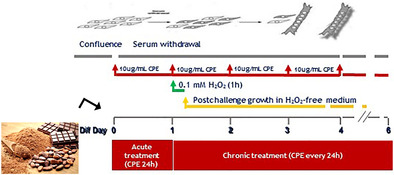

**Summary**

- Phytochemicals like flavonoids, which include subclasses such as flavonols, flavanols, and anthocyanidins, have been shown to impact oxidative stress significantly.
- This research line is promising for developing targeted nutraceutical interventions to aid those seeking to enhance their physical health and performance.

## 1 Introduction

In the dynamic field of muscle regeneration, understanding the interplay between cellular processes and their microenvironments is critical due to the detrimental role of oxidative stress. Muscles known for their potent ability to regenerate can be severely affected by the uncontrolled production of reactive oxygen species (ROS), which include hydrogen peroxide (H₂O₂). These ROS are the mediators of the essential oxidative stress pathway, in which the balance of regenerative and degenerative signals within the muscle is disrupted (Barbieri and Sestili 2012).

In clinical settings, oxidative stress is often observed in conditions like sarcopenia‐muscle degeneration due to aging‐ and in various muscle diseases. ROS generation is accelerated in aging muscle tissues, which has a range of harmful consequences due to the oxidation of DNA, proteins, and lipids, impairing vital cellular processes such as myogenic differentiation. These disruptions are critical since myogenic differentiation is essential for adequate muscle repair and regeneration (Chen et al. 2022).

The overproduction of ROS disrupts the delicate balance within the cellular microenvironment of skeletal muscles, inhibiting critical regenerative processes. Elevated ROS levels can inhibit the Akt/mTOR pathway, a crucial signaling route for protein synthesis and muscle cell growth, impairing the body's ability to repair and regenerate muscle tissue effectively (Sallam and Laher 2016).

Moreover, the regenerative capability of muscle is not solely dependent on biochemical signals but is also influenced by the mechanical environment of the cells. Recent studies have shown that mechanical cues such as substrate stiffness and mechanical forces significantly influence muscle stem cell (MuSC) behavior, affecting their proliferation and differentiation abilities, which are crucial for muscle regeneration (Pang et al. 2023).

Considering the significant impact of oxidative stress on muscle regeneration, therapeutic strategies aimed at decreasing ROS levels or enhancing the antioxidant defenses of muscle cells may be beneficial. Antioxidant approaches, mainly through diet and food supplements, may rebalance the redox state in aging muscles, potentially improving muscle function and enhancing the regenerative capacity.

The influence of diet on muscle regeneration through the modulation of oxidative stress has been investigated. Phenolic compounds found abundantly in fruits, vegetables, teas, and other plant‐based foods are known for their potent antioxidant properties. These compounds may help mitigate oxidative stress and play a crucial role in protecting against cellular damage and supporting muscle health (Kruk et al. 2022).

Moreover, incorporating specific phytochemicals like flavonoids, which include subclasses such as flavonols, flavanols, and anthocyanidins, has been shown to impact oxidative stress significantly (Farzaei et al. 2019; Zeka et al. 2017). These substances effectively reduce oxidative damage that can impair muscle regeneration (Rojano‐Ortega et al. 2003; Jung 2023). Diets rich in these compounds support the body's antioxidant capacity, enhancing muscle recovery and overall health (Petrella et al. 2021; Venderley and Campbell 2006; Vanacore et al. 2018).

Flavonoids are prevalent in various foods, with cocoa being one of the primary sources of flavanols. Cocoa flavanols, including epicatechin, perform their biological activities mainly by acting as antioxidants in the body. These compounds scavenge free radicals and chelate metal ions, preventing cell oxidative damage. In addition, they favor the body's defense antioxidant mechanism, increasing the activity of antioxidant enzymes like superoxide dismutase, catalase, and glutathione peroxidase. Epicatechin, in particular, may provide blood cell protection in other ways by exerting numerous effects, such as enhanced endothelial function, at least in part due to nitric oxide production. Additionally, epicatechin acts as an anti‐inflammatory molecule by suppressing the phosphorylation of NF‐κB, a critical pro‐inflammatory protein, leading to decreased expression of pro‐inflammatory cytokines and adhesion particles (Corr et al. 2021).

In this study, we have explored the effects of a novel cocoa‐rich polyphenol extract (CPE) on the myogenic index, a quantitative measure of myogenic differentiation, employing morphological, ultrastructural, and molecular analyses.

## 2 Materials and Methods

### 2.1 Cocoa Extract Preparation and Analyses

High‐flavanol cocoa was sourced, with the cocoa displaying the highest flavanol content selected for extraction based on analyses performed using the method established by Alañon et al. Briefly, cocoa powders underwent a defatting process involving successive washes with hexane (1:10 w/v) in an ultrasonic bath, followed by centrifugation at 10,000 rpm for 10 min at 4°C. This process was repeated three times to ensure thorough lipid removal. The defatted pellets were then air‐dried for 24 h and freeze‐dried. Polyphenolic extraction was carried out using 70% acetone (1:10 w/v) in an ultrasonic bath, with supernatants recovered post‐centrifugation at 10,000 rpm for 10 min at 25°C and concentrated utilizing a rota‐evaporator (García‐Merino et al. 2022). Extracts were stored at −80°C until further analysis.

Chemical analyses were performed as previously described. Briefly, polyphenolic compounds were analyzed using ultra‐high performance liquid chromatography (UHPLC) coupled with tandem photodiode array detection and electrospray ionization (ESI) triple quadrupole mass spectrometry (MS/MS) on a Xevo TQS system (Waters Corp., Wexford, Ireland). Separation was exerted using a C18 column (150 mm × 2.1 mm, 1.7 μm, Agilent Technologies) at 30°C. The elution utilized a gradient of acidified water (Solvent A) and acetonitrile (Solvent B), with specific gradients detailed for optimal separation. Ionization conditions were optimized for negative ion mode, including a capillary voltage of 2.5 kV and a desolvation temperature of 400°C. Multiple reaction monitoring mode was employed for quantification, using standard mixtures for calibration. Data acquisition and processing were conducted using MassLynx v. 4.1 software (Waters Corp.). The degree of polymerization of cocoa polyphenols was determined via a phloroglucinolysis method, as previously described (García‐Merino et al. 2022).

#### 2.1.1 Study Design and Cell Culture

This study investigated the effects of CPE on myogenic differentiation in C2C12 murine myoblasts exposed to oxidative stress induced by H₂O₂. C2C12 cells, a murine myoblast cell line, were cultured under standard conditions ingrowth medium consisting of Dulbecco's Modified Eagle Medium (DMEM) supplemented with 10% fetal bovine serum (FBS) and 1% penicillin–streptomycin. Cells were maintained at 37°C in a humidified atmosphere containing 5% CO_2_.

#### 2.1.2 Experimental Treatments

Confluent at 80%, the cells underwent serum withdrawal to induce differentiation. The myogenic differentiation process was observed at early (Day 1, D1) and late phases (Day 4, D4, and Day 6, D6) of differentiation. Upon the differentiation commencement, cells were exposed to 0.1 mM H₂O₂ for 1 h at 24 h post‐differentiation to induce oxidative stress with DMEM 0% FBS. To evaluate the protective function of CPE, the cells were treated with 10 μg/mL CPE immediately added upon matrix differentiation (D0). The cells were refreshed with fresh medium every 2 days, and CPE was added every 24 h to avoid degradation of the polyphenols in the extract and ensure chronic exposure. The control groups consisted of cells without treatment, CPE alone, and H₂O₂ alone.

#### 2.1.3 Assessment of Myogenic Differentiation

The myogenic index quantitatively determined myogenic differentiation: the share of nuclei in the myotubes in the total number of myonuclei. May‐Grunwald‐Giemsa‐stained nuclei were observed at D4 and D6. The myogenic index was determined in five fields in a 60 mm dish for each kind of treatment in the microscope, and the number of cells was calculated at 300–500 nuclei per area.

### 2.2 Mitochondrial Morphology and Function Analysis

As mitochondrial function is necessary for myogenesis, MitoTracker Red CMXRos (ThermoFisher, M7512) staining was employed to determine mitochondrial morphology and oxidative challenge with or without CPE treatment. After the treatment, the cells were incubated with 200 nM MitoTracker Red CMXRos at 37°C for 30 min. After incubation, the cells were examined under a Leica TCS SP5 II confocal microscope (Leica Microsystem, Germany). The images captured on D1 and D6 were processed and analyzed using the NIH‐Image J software (National Institutes of Health, Bethesda, MD). The form factor (FF) was calculated from the area (Am) and perimeter (Pm) using the following formula: FF = Pm2/4πAm. FF has the minimal value of 1 when the measured organelle is drawn as a perfect circle (Ananthanarayan, Caprini, and Kubis 2014). FF values > 1 indicate progressive increased mitochondria length and shape complexity.

### 2.3 Transmission Electron Microscopy (TEM)

Further ultrastructural analysis was performed using TEM to evaluate the detailed mitochondrial morphology and to identify signs of cytoprotective effects provided by CPE against H₂O₂‐induced damage. Specifically, the study focused on mitochondrial swelling, cristae organization, and autophagic vacuoles.

C2C12 cells were washed and fixed with 2.5% glutaraldehyde in 0.1 M in phosphate buffer (pH 7.3) for 1 h. The samples were post‐fixed in 1% OsO4 for 1 h, alcohol dehydrated, and embedded in araldite (Salucci et al. 2017). For ultrastructural analysis, thin sections were stained with UranyLess and lead citrate and analyzed using a Philips CM10 transmission electron microscope (FEI Italia SRL, Milano, Italy).

### 2.4 Statistical Analysis

Data were presented as mean ± standard deviation (SD). The statistical significance between groups was evaluated with ANOVA followed by post hoc tests to compare some specific groups. A *p*‐value of < 0.05 was considered statistically significant.

## 3 Results

### 3.1 Cocoa Polyphenol Extract Characterization

As previously reported, the characterization of the CPE confirmed a notable flavanol profile. High‐flavanol cocoa (83 mg/g) was purchased from Chococru (London, UK). The polyphenolic characterization of high‐flavanol cocoa has been described previously (García‐Merino et al. 2022); the main constituents include procyanidin B2 (68 mg/g), (−) epicatechin (8 mg/g), catechin (3.85 mg/g), and procyanidin B1 (3.34 mg/g).

### 3.2 Myogenic Differentiation

**FIGURE 1:**
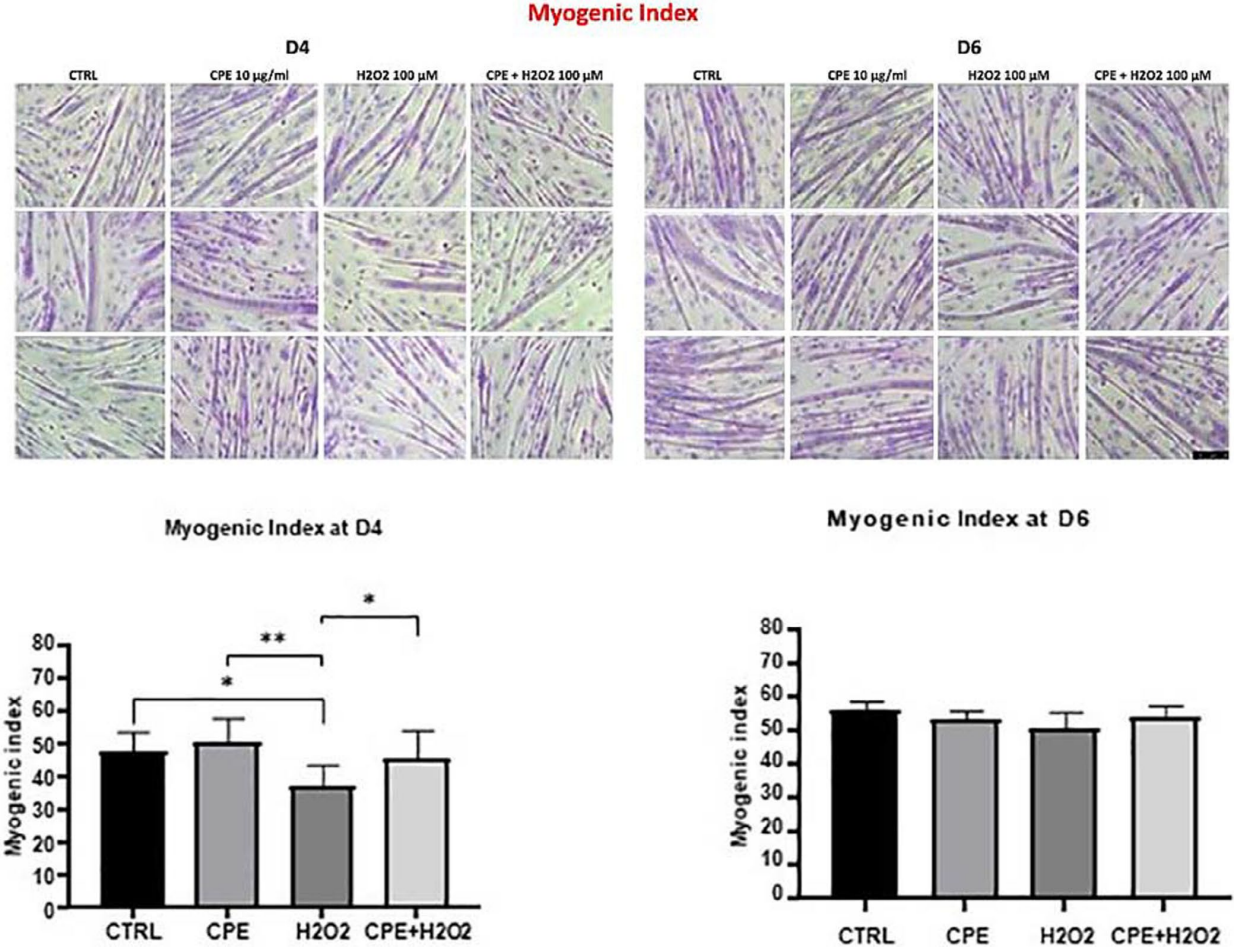
The Myogenic Index was assessed using May‐Grunwald‐Giemsa. Five fields/60 mm dish (*n* = 3) were observed, total no. of nuclei analyzed was 300–500/field. Bar = 50 μm.

The myogenic index assessed at D4 and D6 indicated partial protection of C2C12 cells by CPE from H₂O₂‐induced inhibition of myogenesis. The quantitative assessment through May‐Grunwald‐Giemsa staining provided evidence of CPE's effectiveness in preserving myogenic capacity in the face of oxidative insult, as shown in Figure 1.

### 3.3 Mitochondrial Morphology and Function Analysis

**FIGURE 2:**
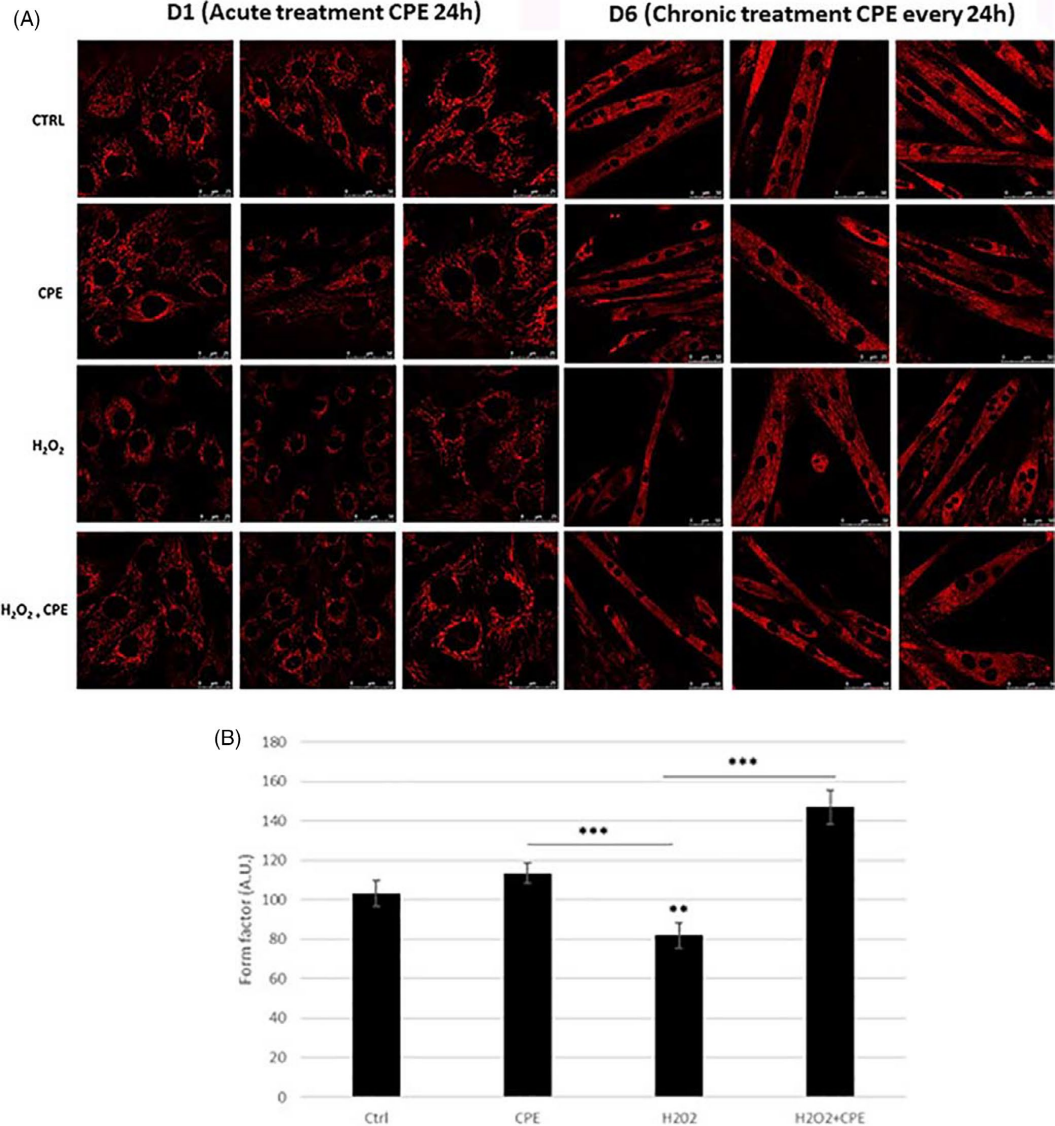
(A) Confocal microscopy of C2C12 myoblasts induced to the myogenic program after the acute (D1) and chronic (D6) CPE treatments after staining with MitoTracker Red CMXRos showed the mitochondrial network morphology and the effect of oxidative challenge with or without CPE supplementation. Bar = 50 μm. Micrographs were taken at D1 and D6. (B) Analysis of confocal images showing the quantification of mitochondrial mass. Graphical representation of morphological meaning of the form factor (FF).

We analyzed mitochondria morphology by confocal microscopy with MitoTracker Red CMXRos labeling to characterize mitochondria status. As shown in Figure 2A in non‐differentiated C2C12 cells (D1 phase), we observed an apparent reduction in mitochondrial mass, which appeared smaller and fragmented in H_2_O_2_‐treated cells compared to the control condition. On the contrary, after acute CPE treatment, the mitochondria appeared more elongated, showing a tubular morphology like that of the control condition (Figure 2A, D1). This effect is also confirmed after chronic treatment D2 phasein which mitochondria of differentiated myoblasts appeared more preserved with a parallel and organized distribution in the CPE‐treated groups compared to the H₂O₂‐group. Morphological changes were quantified in confocal microscopy, measuring the FF parameter, which reflects mitochondria's complexity and branching aspects. After H_2_O_2_ treatment, there was a significant reduction in the FF compared to the control condition (Figure 2A, D6), indicating fragmented and discontinuous mitochondria. Treatment with CPE significantly improved the FF parameter (Figure 2B; *p* ≤ 0.001 vs. H202), suggesting a better‐preserved mitochondria morphology and function.

#### 3.3.1 Transmission Electron Microscopy (TEM)

**FIGURE 3:**
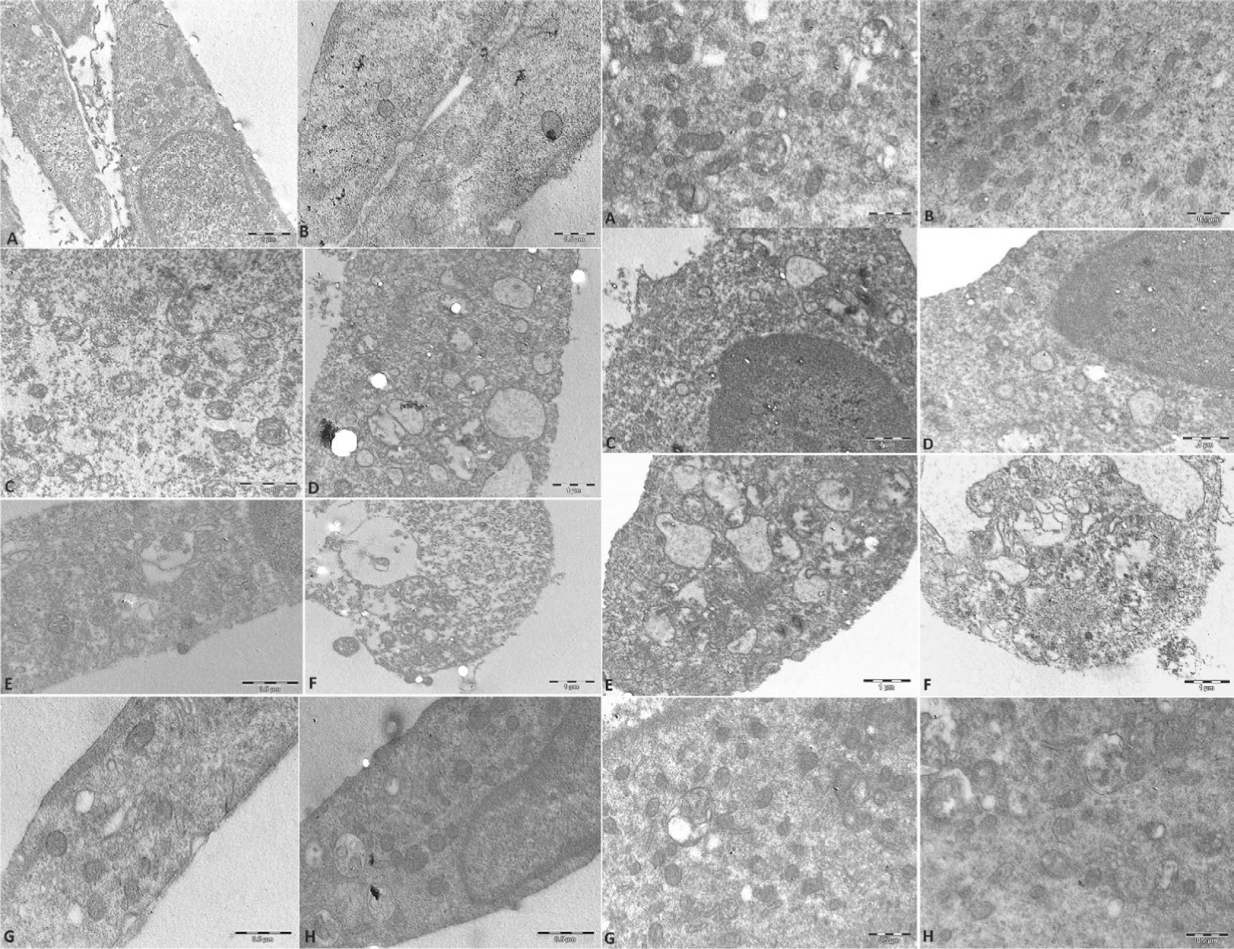
TEM of cells at D1 panel left and D6 panel right. TEM of control cells show a good morphology of mitochondria at D1 (A, B) panel left and D6 panel right (A, B). Also after CPE treatment the cells have good morphology and conserved mithocondria at D1 (C, D) panel left and D6 (C, D) panel right. H_2_O_2_‐treatment induced necrosis; the inserts in the TEM images of H_2_O_2_‐treated cells highlight the mitochondrial swelling and disruption caused by oxidative stress (E, F). CPE prevented the effect of H_2_O_2_ and cells appeared comparable to the control ones, with a higher number of mitochondria. TEM analyses showed a relevant decrease of cytoplasmic vacuolization as well as preserved mitochondrial morphology (G, H) also at D1 and D6. The Bar scale are present on the images.

EM analysis further substantiated the confocal microscopy findings by revealing more detailed structural preservation in CPE‐treated cells. The TEM images highlighted reduced cytoplasmic vacuolization and better conservation of mitochondrial cristae in CPE‐treated groups compared to the H₂O₂ group. This provided ultrastructural evidence of CPE's protective effects against oxidative damage, as illustrated in Figure 3. The effect of CPE is similar in undifferentiated D1 and differentiated D6 cells.

## 4 Discussion

The findings of this research highlight the potential of CPE in safeguarding against oxidative stress‐induced challenges in myogenic differentiation. Given this evidence, it is tempting to speculate that CPE saves the mitochondrial network and maintains a floor of myogenesis under stress conditions. This aligns with flavanols' well‐documented antioxidant and cytoprotective properties, observed in different cellular models (Al‐Dashti et al. 2018) and confirmed in various clinical trials (Massaro et al. 2019).

Significantly, our data indicates that treatment with CPE increases the number and health of mitochondria, which are essential for energy production and cellular functions and crucial for muscle differentiation and recovery. These effects may result, in part, from the flavanols' antioxidant activities (Remels et al. 2014).

In addition, the observed reduction in cytoplasmic vacuolization and the preservation of myogenic capacity with CPE treatment suggest a phytocomplex ability to counteract oxidative stress. Several studies demonstrated that cocoa flavanols, particularly epicatechin, induce cellular antioxidant defenses partially through mechanisms involving the Nrf2 signaling pathway activation (Cordero‐Herrera et al. 2015; Lan et al. 2017).

Indeed, a connection between the activation of the Nrf2 pathway and morphological changes in muscle cells, particularly under conditions of oxidative stress, was observed. Morphological changes observed with the activation of Nrf2 include alterations in mitochondrial structure and distribution. This is significant because mitochondria are critical for energy production in muscle cells, and their dysfunction can lead to various muscular diseases. Studies have reported that activation of Nrf2 can lead to improved mitochondrial biogenesis and resistance to oxidative damage (Duleh et al. 2016). Also, changes in the structure and function of the sarcoplasmic reticulum can affect muscle contraction and overall muscle function, with potential implications for muscle fatigue and endurance (Ángel García‐Merino et al. 2020).

A nuanced action of cocoa flavanols in muscle health, partially salvaged myogenic differentiation by CPE, could be beneficial in elevated stress markers in post‐exercise recovery or muscle diseases with the complicating role of oxidative stress in the pathology. Procyanidin B2 has been shown to induce the expression of genes associated with slow‐twitch fibers through the activation of AMPK signaling. Slow‐twitch fibers are characterized by their high resistance to fatigue and reliance on oxidative metabolism (Xu et al. 2022).

Research on the beneficial effects of cocoa flavanol intake after exercise showed mixed results. The possible reason for various outcomes may be the intake of cocoa products containing different amounts of flavanols and doses. However, some data suggest that flavanol consumption can significantly reduce oxidative stress markers, favoring recovery, provided with an appropriate period and adequate intake. For instance, Corr et al. found a decrease in oxidative stress markers, such as malondialdehyde (MDA), after 2 weeks of routine CF intake while exercising (Corr et al. 2021). Other studies have found minimal or no significant effects of acute cocoa flavanol intake on exercise recovery or performance. A study on the acute post‐exercise consumption of cocoa‐based beverages with varying flavanol content did not find significant benefits in reducing muscle soreness or improving recovery markers such as creatine kinase levels (Peschek et al. 2013). On the contrary, a study conducted on well‐trained cyclists reported no significant changes in oxidative stress or nitric oxide production with acute CF consumption (Decroix et al. 2017). These findings suggest that while cocoa flavanols have potential health benefits, their effectiveness in enhancing post‐exercise recovery might depend on many factors, including the amount of flavanol concentration in the products, the time, the regularity, and the dosage used (Lee et al. 2006; Ismaeel et al. 2024).

CPE has a total flavanol content of 83.819 mg/g. Considering a serving size that delivers 419.09 mg of flavanols, our extract shows higher flavanol concentration when compared to other cocoa extracts used in clinical trials. For instance, previous studies have reported benefits with sub‐chronic intakes of cocoa flavanols ranging from 168 to 197.4 mg daily over 2 weeks. Another study utilizing 1765 mg of cocoa extract daily for a week before exercise showed positive effects (Corr et al. 2021). In contrast, the serving size of CPE allows the assumption of 419.09 mg of flavanols per serving, which exceeds the amounts in these studies and suggests a higher potential for clinical efficacy (García‐Merino et al. 2022). Specifically, our serving provides a substantial amount of Procyanidin B2 (343.54 mg), a compound endowed with antioxidative capacity. Given the documented importance of dosing in achieving significant clinical outcomes, the higher concentration of specific flavanols in our CPE may account for the more pronounced physiological effects observed (García‐Merino et al. 2022). We used a CPE concentration of 10 μg/mL, corresponding to 0.83819 μg/mL of total flavonols, representing the active‐active in vitro within healthy cells. This concentration is within the bioactive range observed in different studies, where flavonoid exposure at similar levels has been associated with antioxidant properties (Li et al. 2022; Zhang et al. 2023; Ayeleso, Oguntibeju, and Brooks 2014).

In addition, concerning the CPE‐induced increase in the number and health of mitochondria, these findings could be particularly significant when considering the bioavailability of flavanols. While specific data on the bioavailability of flavanols is necessary to interpret these results fully, research indicates that even though specific flavanols can be limited, they are nonetheless detectable in plasma after consumption and can exert systemic effects (Rios et al. 2003; Holt et al. 2002; Natsume et al. 2023). Several studies demonstrate this phenomenon. The absorption of cocoa flavanols allows their entry into the human circulatory system. In particular, monomeric and dimeric flavanols, like those found in CPE, remain intact through gastric digestion and reach the upper intestine, where they are adsorbed and stay in plasma within 30 min to an hour after ingestion (Kwik‐Uribe and Bektash 2008).

Morphological studies have afforded valuable insights into the impact of CPE on muscle cell structures. These changes, which include improved mitochondrial integrity and function and reduced cytoplasmic vacuolization, underscore the potential of CPE in promoting muscle health.

## 5 Conclusions

The effect of CPE in this oxidative stress paradigm is the expression of its cytoprotective activity on multiple targets relevant to myogenesis rather than the result of a single, precise mechanism. In this scenario, CPE mitochondrial effects are likely to represent essential players. This data also strengthens the notion that, at nutritionally attainable concentrations, CPE exerts antioxidant/cytoprotective activity in C2C12 cells and rescues their morphology.

These findings suggest dietary recommendations incorporating CPE could benefit muscle recovery and overall health. Further research on identifying the pathways involved and the interaction between nutritional flavanols and muscle cell regulators will allow us better to understand CPE's role in muscle health and recovery. This line of research is promising for developing targeted nutraceutical interventions to aid those seeking to enhance their physical health and performance.

## Author Contributions

**Jose Angel Garcia‐Merino:** conceptualization, data curation. **Vittoria Carrabs:** investigation. **Fabio Ferrini:** methodology, software. **Sara Salucci:** conceptualization, methodology, writing – original draft. **Michela Battistelli:** conceptualization, methodology, writing – original draft, supervision, data curation. **Sabrina Burattini:** writing – review and editing, investigation. **Francesca Luchetti:** investigation, formal analysis. **Maria Gemma Nasoni:** data curation, software. **Giosuè Annibalini:** project administration, visualization, writing – review and editing. **Matteo Micucci:** conceptualization, writing – original draft, funding acquisition, project administration. **Federico Gianfanti:** validation, formal analysis. **Piero Sestili:** supervision, methodology, conceptualization, writing – review and editing, project administration. **Mar Larrosa:** funding acquisition, project administration, writing – review and editing. **Elena Barbieri:** project administration, resources, conceptualization, writing – original draft.

## Conflicts of Interest

The authors declare no conflicts of interest.

## Data Availability

The data that support the findings of this study are available from the corresponding author upon reasonable request.
